# Effect of social deprivation on blood pressure monitoring and control in England: a survey of data from the quality and outcomes framework

**DOI:** 10.1136/bmj.a2030

**Published:** 2008-10-28

**Authors:** Mark Ashworth, Jibby Medina, Myfanwy Morgan

**Affiliations:** 1 King’s College London, Department of General Practice and Primary Care, Division of Health and Social Care Research, London SE11 6SP; 2 King’s College London, Department of Public Health Sciences, Division of Health and Social Care Research, London SE1 3QD

## Abstract

**Objective** To determine levels of blood pressure monitoring and control in primary care and to determine the effect of social deprivation on these levels.

**Design** Retrospective longitudinal survey, 2005 to 2007.

**Setting** General practices in England.

**Participants** Data obtained from 8515 practices (99.3% of all practices) in year 1, 8264 (98.3%) in year 2, and 8192 (97.8%) in year 3.

**Main outcome measures** Blood pressure indicators and chronic disease prevalence estimates contained within the UK quality and outcomes framework; social deprivation scores for each practice, ethnicity data obtained from the 2001 national census; general practice characteristics.

**Results** In 2005, 82.3% of adults (n=52.8m) had an up to date blood pressure recording; by 2007, this proportion had risen to 88.3% (n=53.2m). Initially, there was a 1.7% gap between mean blood pressure recording levels in practices located in the least deprived fifth of communities compared with the most deprived fifth, but, three years later, this gap had narrowed to 0.2%. Achievement of target blood pressure levels in 2005 for practices located in the least deprived communities ranged from 71.0% (95% CI 70.4% to 71.6%) for diabetes to 85.1% (84.7% to 85.6%) for coronary heart disease; practices in the most deprived communities achieved 68.9% (68.4% to 69.5%) and 81.8 % (81.3% to 82.3%) respectively. Three years later, target achievement in the least deprived practices had risen to 78.6% (78.1% to 79.1%) and 89.4% (89.1% to 89.7%) respectively. Target achievement in the most deprived practices rose similarly, to 79.2% (78.8% to 79.6%) and 88.4% (88.2% to 88.7%) respectively. Similar changes were observed for the achievement of blood pressure targets in hypertension, cerebrovascular disease, and chronic kidney disease.

**Conclusions** Since the reporting of performance indicators for primary care and the incorporation of pay for performance in 2004, blood pressure monitoring and control have improved substantially. Improvements in achievement have been accompanied by the near disappearance of the achievement gap between least and most deprived areas.

## Introduction

High blood pressure is the single most important risk factor worldwide for the development of cardiovascular disease, being responsible for about half of all cases of strokes, ischaemic heart disease, and heart failure.^1^ High blood pressure seems to be a more powerful cardiovascular risk factor than indicated by earlier studies.^2^ The gains achieved by successful blood pressure control (defined as systolic blood pressure ≤140 mm Hg) have been quantified for adults in the United Kingdom.^3^ With an assumed reduction of 28-44% in stroke and 20-35% in ischaemic heart disease, a total of 43 000 strokes and 83 000 cases of ischaemic heart disease would be prevented in the UK annually. However, gains in health outcomes must be set against the considerable costs involved.

Over the past two decades, several countries have conducted community surveys on blood pressure detection and control. Based on national surveys, 12-25% of the population of five European countries had blood pressure above 160 mm Hg systolic or 90 mm Hg diastolic compared with 7.4% in Canada and 5.3% in the United States.^4^ Among these hypertensive populations, 41-52% of those in European countries were taking antihypertensive treatment compared with 62% in Canada and 78% in the US. The authors commented that European countries had higher hypertension prevalence and substantially lower treatment rates coupled with less successful control of hypertension.

In England the most recent detailed national population survey data are from the health survey for England conducted in 2003. A total of 8834 adults aged ≥16 years were surveyed with blood pressure readings taken on three occasions. Their mean blood pressure was 131/75 mm Hg. Of those with hypertension (blood pressure ≥140/90 mm Hg or taking antihypertensive drugs), 61.7% were aware of their diagnosis, 47.7% were receiving antihypertensive treatment, and 21.8% had adequate blood pressure control (blood pressure ≤140/90 mm Hg).^5^

In 2004 the contract for all UK general practitioners was revised to include a pay for performance system accounting for up to a quarter of total annual income. This system, termed the quality and outcomes framework (QOF), has provided new performance data aggregated at practice level.^6^ A total of 135 performance indicators are currently applied to each practice, measuring aspects of the structure, processes, and outcomes of primary care. Many of the performance indicators have a public health dimension, reflecting the increased public health role of primary care. Achievement of blood pressure recording for all registered patients aged ≥45 years is monitored annually. So too is the achievement of blood pressure target levels for all patients with any of five chronic conditions that are included in the QOF—hypertension, coronary heart disease, stroke and transient ischaemic attacks, diabetes, and chronic kidney disease.

The linkage of financial incentives to performance targets was intended to drive up the standards of primary care. It has also been observed that health inequalities between different population groups may be diminished as overall collective achievement increases. For example, the introduction of cervical smear targets for UK general practices in 1990 resulted in practices in affluent areas rapidly achieving the highest targets, initially widening the health inequality gap. Within a few years, however, practices in deprived areas had caught up, resulting in an eventual reduction in inequality based on social deprivation.^7^ This phenomenon has been termed the inverse equity hypothesis.^8^ In general terms, this hypothesis predicts that new public health interventions initially reach the wealthier sector of the population and then later begin to benefit the poor. In consequence, inequity ratios are initially increased, only to be diminished once the poor gain access to the intervention and a ceiling effect is reached in the richer population. Reduced QOF achievement was reported for practices in socially deprived areas in the first year of the QOF, though differences were small (6.1% of the total QOF score), but by year 2, this gap had reduced to 2.9%.^9^ No studies have yet reported on year 3 QOF data, and none has explored the possible association between the achievement of blood pressure indicators and social deprivation.

We decided to use the data available through the QOF to provide a current perspective on two measures—national rates of blood pressure monitoring in primary care patients and the achievement of blood pressure control targets in patients with chronic conditions. We aimed to describe changes in these two measures over the three years since QOF data have been available. In addition, we describe the effect of any changes over time on health inequalities between general practices in deprived and less deprived communities.

## Methods

### Quality and outcomes framework data

We obtained QOF data covering the three years from April 2004 to March 2007 for all general practices in England. Data for the preceding year were collected in February (prevalence data) and March (performance indicator data) of years 2005, 2006, and 2007.^10^ We confined our analysis to the following:

- The proportion of all registered patients aged ≥45 years who had had their blood pressure taken within the preceding five years
- The prevalence of five chronic conditions (hypertension, coronary heart disease, stroke and transient ischaemic attacks, diabetes, and chronic kidney disease) and the achievement of blood pressure targets for each of these conditions.

The denominator for the analysis of all QOF data was the total population of patients on the respective disease registers for each practice. This method of analysis precludes the process of “exception reporting,” whereby general practitioners are permitted to omit certain patients from their performance data on the grounds of unsuitability (for example, patients who are already receiving the maximum tolerated hypotensive drug dose, who fail to attend the surgery in spite of three invitations, or who are terminally ill). For a pay for performance scheme, exception reporting is an important means to avoid penalising a general practitioner when patient-dependent factors, beyond the control of the general practitioner, diminish the achievement of clinical indicators. For research purposes, exception reporting introduces bias, since different general practitioners have different exception reporting thresholds.^11^ Although publicly available QOF data do not allow the proportion of exception reporting to be calculated for all clinical indicators, it was possible to make this calculation for each of the QOF indicators included in our study. All data presented in this study are the values reported by general practitioners before they had excluded any patients using the mechanism of exception reporting.

### Practice and population characteristics

We obtained a detailed summary of practice characteristics.^12^ Variables included: practice list size, a breakdown of the registered population by age and sex, the number of full time equivalent general practitioners, and training practice status.

We obtained data from the 2001 national UK census based on the lower layer “super output area” (SOA) for each practice.^13^ Each such area consists of about 1500 people within a defined geographical locality. Census data based on the home address of all patients registered at a general practice are not available in England, so we used the super output area in which a general practice was located as a proxy for the registered population at each practice. The super output area forms the basis for calculating the index of multiple deprivation, 2004, which consists of seven domains of social deprivation, mostly derived from census data in 2001 but with some domains (such as education, housing, and crime) updated more recently.^14^ We therefore obtained and analysed deprivation data at practice level rather than patient level.

Like most commonly used deprivation indices, the index of multiple deprivation does not include a measure of ethnicity. These data are available from the 2001 national census, and we added them to our own dataset, again aggregated at the level of super output area.

### Statistical methods

We constructed a dataset for all general practices in England containing data from the QOF, the practice, and census data for the surrounding super output area. We removed from the dataset practices that were no longer independent at the end of the study year or had a list size of <750 patients or <500 per full time general practitioner, on the grounds that these practices were likely to be newly formed or about to be closed. On this basis, we removed 61 practices in year 1, 145 in year 2, and 180 in year 3. Postcode and super output area anomalies meant that we were unable to match deprivation and ethnicity data for 35 practices in year 1, none in year 2, and 361 in year 3. The final dataset consisted of 8515 practices (99.3% of the total) in year 1, with 8480 of these practices linked to the 2004 index of multiple deprivation; 8264 practices (98.3% of the total) in year 2, all linked to the deprivation index; and 8192 (97.8% of the total) in year 3, with 7831 linked to the deprivation index.

Firstly, we explored the relationship between social deprivation and the achievement of the six QOF indicators related to blood pressure that we included in our study. We conducted this analysis by comparing the achievement of practices located in the most deprived fifth of super output areas in the country with the achievement of practices in the least deprived fifth. We calculated mean values for each of the blood pressure related variables in all practices and then recalculated them as weighted means (based on the number of registered patients) in order to adjust for the effect of practice size. To enable comparison of mean values in the most deprived and least deprived areas, we estimated the 95% confidence intervals of the means. We then searched for possible confounding variables using multivariate analysis and a forward stepwise method; variables were entered into the equation if the significance of the association on univariate analysis, P, was <0.1.

## Results

### Practice characteristics

Table 1 shows the characteristics of the practices in the least deprived and most deprived fifths of the super output areas. Although there are about twice as many practices in the deprived areas, they have larger list sizes per full time equivalent general practitioner and are less likely to be training or group practices.

**Table 1 tbl1:** Characteristics of general practices in the least deprived and most deprived fifths of the “super output areas”* in England during the first three years of the quality and outcomes framework

Characteristic	Least deprived areas				Most deprived areas		
	Year 2004-5	Year 2005-6	Year 2006-7		Year 2004-5	Year 2005-6	Year 2006-7
No of practices	1096	1228	1170		2391	2373	2225
No of full time equivalent general practitioners	3974	4660	5617		6036	6342	7427
Mean list size per full time equivalent general practitioners	2098	2063	1694		2285	2284	1982
% of practices that were single handed	14.4	13.4	8.9		36.2	35.1	28
% of practices that were training practices	38.0	38.2	38.2		19.7	19.1	19.8

*See text and National Statistics^13^ for explanation of super output areas.

### Blood pressure recording in the adult population

Table 2 shows the proportions of all registered patients aged ≥45 years with a record of a blood pressure reading in the preceding five years. The table shows data for general practices in the most and least deprived fifths of the super output areas and shows both the mean practice scores and the mean scores weighted by practice list size. The small discrepancy between values for blood pressure recording in the least and most deprived areas in 2005 was dwarfed by the overall improvement in values over the three years. Moreover, by 2007, the difference between the general practices in the least and most deprived areas had all but disappeared.

**Table 2 tbl2:** Monitoring of blood pressure of adult patients (≥45 years) registered with general practices in the least deprived and most deprived fifths of the “super output areas”* in England. Values are mean (95% CI) percentage of patients in each practice with blood pressure recorded within the past 5 years

	Least deprived areas				Most deprived areas		
	Year 2004-5	Year 2005-6	Year 2006-7		Year 2004-5	Year 2005-6	Year 2006-7
Crude values	82.8 (82.3 to 83.2)	86.3 (86.0 to 86.6)	88.2 (88.0 to 88.4)		80.0 (79.5 to 80.5)	82.8 (82.3 to 83.2)	88.1 (87.9 to 88.3)
Weighted values†	82.8 (82.8 to 82.8)	86.0 (86.0 to 86.0)	87.9 (87.9 to 87.9)		81.1 (81.1 to 81.1)	85.5 (85.5 to 85.5)	87.7 (87.7 to 87.7)

*See text and National Statistics^13^ for explanation of super output areas.

†Mean values weighted according to number of patients registered at each practice.

When we analysed data for all practices in England, the mean number of patients with an up to date blood pressure recording in 2007 (year 3 of the QOF) was 88.3% (n=53.21 million); after weighting for practice list size, the mean was 87.9%. Comparable values for 2005 were 81.6% and 82.3% (n=52.78 million), respectively.

### Prevalence of five chronic conditions

Table 3 summarises the crude prevalence reported by practices in the least and most deprived areas for each of the five long term conditions included in our study. Recorded disease prevalence has increased over the three year observation period, but the differences in recorded prevalence between least and most deprived areas are small.

**Table 3 tbl3:** Crude recorded prevalence (%) for five chronic conditions among adult patients (≥45 years) registered with general practices in the least deprived and most deprived fifths of the “super output areas”* in England

Condition	Least deprived areas				Most deprived areas		
	2004-5	2005-6	2006-7		2004-5	2005-6	2006-7
Hypertension	11.4	12.4	12.0		10.5	11.3	12.6
Coronary heart disease	3.2	3.2	3.6		3.6	3.7	3.2
Stroke and transient ischaemic attacks	1.4	1.5	1.5		1.3	1.4	1.6
Diabetes	2.8	3.0	4.1		3.8	4.0	4.1
Chronic kidney disease†	—	—	2.0		—	—	2.4

*See text and National Statistics^13^ for explanation of super output areas.

†Chronic kidney disease data were included in the quality and outcomes framework only in 2006-7.

### Achievement of blood pressure targets for five chronic conditions

Table 4 summarises the success of the practices in the least and most deprived areas at achieving the blood pressure targets incorporated into the QOF. The targets set for diabetes and chronic kidney disease are more demanding than for the other chronic conditions. The values shown are mean values for the proportion of patients at each practice who had their blood pressure successfully controlled at or below the target level. From the baseline set in 2005, there have been substantial improvements in the achievement of blood pressure targets across all the conditions included in our study. Modest shortfalls in blood pressure control by practices in more deprived areas have largely disappeared by the third year of the QOF, even though the small residual differences were significant. Weighting the mean values to account for practice list size produced almost identical results.

**Table 4 tbl4:** Achievement of blood pressure targets set in the quality and outcomes framework for five chronic conditions among adult patients (≥45 years) registered with general practices in the least deprived and most deprived fifths of the “super output areas”* in England. Values are mean (95% CI) percentages of patients at each practice whose blood pressure was within the target value†

Condition	Least deprived areas				Most deprived areas		
	2004-5	2005-6	2006-7		2004-5	2005-6	2006-7
Hypertension	72.4 (71.9 to 72.9)	75.9 (75.5 to 76.3)	78.0 (77.7 to 78.4)		69.1 (68.6 to 69.6)	74.7 (74.3 to 75.1)	77.4 (77.1 to 77.7)
Coronary heart disease	85.1 (84.7 to 85.6)	87.5 (87.2 to 87.9)	89.4 (89.1 to 89.7)		81.8 (81.3 to 82.3)	86.1 (85.8 to 86.4)	88.4 (88.2 to 88.7)
Stroke and transient ischaemic attacks	82.8 (82.2 to 83.3)	85.5 (85.0 to 85.9)	87.5 (87.2 to 87.9)		78.5 (77.9 to 79.1)	83.7 (83.3 to 84.1)	86.2 (85.8 to 86.5)
Diabetes	71.0 (70.4 to 71.6)	74.9 (74.4 to 75.4)	78.6 (78.1 to 79.1)		68.9 (68.4 to 69.5)	74.5 (74.1 to 74.9)	79.2 (78.8 to 79.6)
Chronic kidney disease‡	—	—	89.9 (89.2 to 90.7)		—	—	88.7 (88.1 to 89.4)

*See text and National Statistics^13^ for explanation of super output areas.

†Target blood pressure values were ≤150/90 mm Hg for hypertension, coronary heart disease, and stroke and transient ischaemic attacks; ≤145/85 mm Hg for diabetes; and ≤140/85 mm Hg for chronic kidney disease.

‡Chronic kidney disease data were included in the quality and outcomes framework only in 2006-7.

### Association between blood pressure indicators, social deprivation, and possible confounding variables

We constructed a series of regression models to explore the role of possible confounding variables. For each of the five chronic conditions in our study, none of the models explained more than 2.5% of the variation in achievement of target blood pressures (results available from the authors). However, 6.5% of the variation in the proportion of adult patients with up to date blood pressure monitoring was explained by four factors (table 5). The training status of the practice, its singlehanded status, and disease prevalence values were not significant predictor variables.

**Table 5 tbl5:** Multivariate analysis of differences in the proportion of adult patients (≥45 years) with up to date blood pressure monitoring in general practices in the least deprived and most deprived fifths of the “super output areas”* in England. Results shown only for those variables with significant association

Practice characteristic	Adjusted regression coefficient B (95% CI)	Standardised adjusted regression coefficient β
Proportion of population black†	−0.19 (−0.20 to −0.17)	−0.25 (P<0.001)
No of full time equivalent general practitioners	−0.22 (−0.27 to −0.18)	−0.13 (P<0.001)
List size per full time equivalent general practitioner	0.00 (−0.001 to −0.00)	−0.07 (P<0.001)
Index of multiple deprivation, 2004, score	−0.02 (0.01 to 0.03)	0.06 (P<0.001)

*See text and National Statistics^13^ for explanation of super output areas.

†Defined as black or black British in census data.

## Discussion

### Main findings of this study

Blood pressure monitoring levels of all adult patients (aged ≥45 years) registered at general practices in England have risen by 5% over the three years during which QOF data have been available such that, by 2007, 88% of the adult population have had their blood pressure measured in the preceding five years. The weighted mean, which gives a more accurate indication of the proportion of the national population who have had blood pressure screening, produced an almost identical value.

When blood pressure monitoring data first became available through the QOF dataset, there was a difference in blood pressure monitoring between most and least deprived areas. However, as overall blood pressure monitoring levels increased, this inequality has narrowed over the three years such that, by 2007, the difference was negligible (0.2%).

There are marked differences between practices in least and most deprived areas. For example, deprived areas have more singlehanded practices and less training practices. Regression analysis suggested that neither of these two factors had acted as confounding variables. However, on multivariate analysis (in 2007), practices found to be performing less well in terms of blood pressure monitoring were those with higher proportions of black or black British residents in the local population, were situated in less deprived areas, had larger numbers of general practitioners, and had larger list sizes per general practitioner. Hence, in the third year of QOF, social deprivation was no longer having a negative effect on blood pressure monitoring and, once corrected for confounding, had a weakly positive effect. The strongest confounding effect (based on the standardised regression coefficients) was the proportion of black or black British people in the local community.

We also found high levels of blood pressure control for the five chronic conditions included in our study. For all five conditions, the achievement of target blood pressure targets had improved substantially over the three year study period. The greatest increase over time was observed for blood pressure control in diabetics in the most deprived practices, which improved by over 10%, with 79% achieving target blood pressure levels by 2007.

Just as for overall blood pressure screening in the adult population, the improvements in achievement of blood pressure targets in chronic conditions have resulted in almost complete disappearance of the differences between least and most deprived areas. In 2005, the shortfall in the achievement of blood pressure targets in most deprived areas compared with the least deprived ranged from 2.1% to 4.3%. By 2007, the differences in achievement ranged from 1.3 to −0.6 (mean blood pressure control was slightly better in diabetic patients in deprived areas). We conducted multivariate analysis and constructed models for each of the diseases, but these had low predictive power and added little to the univariate findings. In particular, ethnicity did not emerge as a significant confounding covariable for blood pressure control. This suggests that ethnicity played a role in determining the level of blood pressure monitoring by general practitioners but not in the control of high blood pressure.

### What is already known on this topic?

Data from the health survey for England have shown steady improvements in population blood pressure control over the decade 1994-2003.^5^ However, our own results suggest that rapid improvement has occurred in the past three years and that overall population levels of blood pressure control are now likely to be better than those derived from the 2003 health survey for England. Less clear is the improvement in blood pressure detection and control by general practices in England before 2004, when national data did not exist. There is evidence of substantial improvement in certain blood pressure indicators in primary care before the introduction of QOF. One study of 60 practices found that the proportion of patients with coronary heart disease and a blood pressure of ≤150/90 increased from 47.3% in 1998 to 72.2% in 2003.^15^

The inverse equity hypothesis describes trends in health inequalities during a period of rapid overall improvement in standards.^8^ Our findings add to the observations of others that performance indicators for health care are likely to result in improved achievement of those items that are linked to incentives and, at the same time, narrow health inequalities.^8^ The hypothesis also describes initial increases in health inequality during a process of change, followed by a period of convergence. Our data, taken from three time points over three years (reporting takes place on the final day of March, each year) were probably too infrequent to observe more complex trends taking place over short time periods.

### What this study adds

Based on a national study, we have presented the first report of rapidly rising achievement of blood pressure monitoring and control in primary care over recent years and the narrowing of the small but real initial differences based on social deprivation, in terms of these blood pressure variables. Although no comparable national data are available that predate the QOF era, it is likely that pay for performance incentives have contributed to these overall improvements.

This study, although based on aggregated data, provides a summary of recorded blood pressure variables for 53 million people. As such, it offers a far more comprehensive survey than data obtained from population surveys of volunteers.

Recent calls for the introduction of five-yearly cardiovascular screening for all adults in the UK aged 45-74 years have highlighted the national importance of preventing cardiovascular disease, which is now seen as a government priority.^16^ QOF data provide a national perspective on one risk factor for cardiovascular disease but do not provide similar population data on other risk factors.

### Limitations of this study

The values for successful blood pressure control obtained in our study are much higher than previously reported from population surveys in the UK.^5^ In part, this may be attributable to rapid recent improvements. The results were not subject to bias arising from the process of exception reporting.^11^

Our results may have overestimated success because the practice disease register is not validated. Indeed, prevalence figures reported by general practices are lower than predicted by community surveys. For example, the 2003 health survey for England found a national crude prevalence for coronary heart disease of 7.4% in men and 4.5% in women,^17^ and a UK database of more than two million general practitioner case records found a crude adult prevalence for coronary heart disease of 4.0%.^18^ Compared with the QOF’s figure for coronary heart disease prevalence of 3.5%, both these surveys imply incomplete case finding in QOF. This discrepancy is particularly noticeable for chronic kidney disease, with the QOF prevalence figures being much lower than predicted, and general practitioners may be reporting only those patients whose blood pressure is more readily controlled.^19^ By undercounting patients with each given disease, success at achieving national public health targets will be correspondingly decreased. Our own data suggest that case finding for hypertension was hampered at first in deprived areas, but by the third year of QOF, the prevalence of hypertension recorded by general practitioners was slightly higher in deprived areas. Hypertension is probably more prevalent in deprived areas,^20^ although not all studies have found a social gradient.^21^

Black patients are often not well engaged with primary care in the UK, but this is the first report that blood pressure screening might be reduced in this patient group.^22^ Other studies have reported relatively high detection rates for hypertension in black patients.^23^ Our data do not, however, establish a causative relation because the observed association was between lower blood pressure screening rates in practices located in areas with higher black populations; there were no direct ethnicity data for the people attending general practices. No association was observed between South Asian ethnicity and levels of blood pressure recording. Similarly, the lack of ethnicity data about those with established hypertension limits the interpretation of our finding that the ethnicity of the local population was unrelated to success at blood pressure control, whereas others have noted that blood pressure control is poorer in hypertensive patients from an ethnic minority.^23^ ^24^

Several general limitations affect the interpretation of QOF data. QOF was not designed as a research tool, and the data are not validated externally. QOF data may be presented in a more favourable light in order to maximise practice income. For example, patients with poorly controlled blood pressure may not appear on the disease register or may have their blood pressure recorded in “free text” rather than in a coded format and thus be invisible to QOF; they may be defined as “unsuitable” and thus excluded by exception reporting; or they may be included but recorded with an optimistic blood pressure reading based on non-standard procedures. In spite of these temptations, and in the knowledge that QOF data are subject to annual scrutiny by health service managers, Doran et al concluded that the overall level of gaming was low with a median exception reporting rate of 6%, although a small number of practices were characterised by high exception reporting, and some aspects of gaming remain hard to quantify.^11^

The level of scrutiny by health service managers may itself introduce bias into the recording of QOF achievement (including blood pressure targets) since data verification methods differ between localities, some practices will be the subject of far greater scrutiny than others, and new audit procedures have been introduced since the early years of QOF. Prevalence data available in the QOF database are not standardised for age and sex. Individual patient data are not available through QOF, and many patients appear on more than one of the disease registers at the same time.

General practice postcodes were used as a proxy for the postcodes of registered patients at each practice. Although this makes our data susceptible to the ecological fallacy, a primary care study in Rotherham concluded that deprivation scores linked to practice postcodes did provide a valid proxy for patient-level deprivation measures and may even underestimate the association between deprivation and all cause mortality.^25^

Finally, our survey was not able to obtain consultation data. We therefore have no information about how the blood pressure recordings of adult patients were interpreted or how effectively they were used in the diagnosis and treatment of hypertension. Moreover, the blood pressure readings presented by practices for QOF may not be representative of the average blood pressure readings taken over the preceding year, since only the final blood pressure reading recorded during the QOF year is included in the QOF dataset. Similarly, the lack of consultation data means that blood pressure recording can be analysed only according to the timeframes of QOF itself. This results in inconsistencies such that well controlled blood pressure is defined as within the previous 15 months for patients with coronary heart disease but within the previous nine months for hypertensive patients. Blood pressure monitoring of the adult population aged ≥45 years can be analysed only to determine if one or more blood pressure readings have been recorded over the preceding five years, and detailed analysis of more recent data is not possible.

### Conclusion

Performance indicators relating to the detection and control of blood pressure were introduced into primary care in England in 2004. Since then, blood pressure monitoring and control have improved substantially. Improvements in achievement have been accompanied by the near disappearance of the achievement gap between least and most deprived areas.

**What is already known on this topic**

High blood pressure is the single most powerful risk factor for the development of cardiovascular disease
Successful control of blood pressure has the potential to reduce greatly the incidence of cardiovascular disease but is less likely to be achieved in more socially deprived areas

**What this study adds**

Levels of blood pressure monitoring and control in England have increased substantially over the years 2005 to 2007
This increase has been accompanied by narrowing of the small but real social gradients in blood pressure monitoring and control, which were found to have improved more rapidly in more deprived areas
The introduction of a series of primary care performance indicators related to blood pressure control may have contributed to this success

## Notes

We thank Stevo Durbaba for help in preparing the database and Roger Jones for helpful advice on the manuscript.

Ethical approval: Guy’s Research Ethics Committee approved the study.

Provenance and peer review: Not commissioned; externally peer reviewed.

## Notes

**Cite this as: ***BMJ* 2008;337:a2030
